# *Enterococcus mundtii* CRL35 as a Dual-Route Treatment for Cutaneous Leishmaniasis: Preclinical Safety and Efficacy Assessment

**DOI:** 10.3390/tropicalmed11090256

**Published:** 2026-09-10

**Authors:** María A. Occhionero, Daniela A. Gaspar, Daniela E. Barraza, María Elisa Vázquez, Brenda A. Zabala, Rita A. Sandoval, Lucila Saavedra, Cecilia Pérez Brandán, Natalia S. Corbalán, Leonardo Acuña

**Affiliations:** 1 Unidad de Biotecnología y Protozoarios (UBIPRO), Instituto de Patología Experimental “Dr. Miguel Ángel Basombrío” (IPE), Consejo Nacional de Investigaciones Científicas y Técnicas (CONICET), Universidad Nacional de Salta, Salta A4400, Argentina; mariaocchionero@conicet.gov.ar (M.A.O.); danielagaspar@conicet.gov.ar (D.A.G.); barrazadaniela@conicet.gov.ar (D.E.B.); elisa.vazquez@conicet.gov.ar (M.E.V.); b.zabala@conicet.gov.ar (B.A.Z.); cecilia.perezbrandan@conicet.gov.ar (C.P.B.); 2 Laboratorio de Biotecnología Microbiana, Escuela de Biología-Facultad de Ciencias Naturales, Universidad Nacional de Salta, Salta A4400, Argentina; ritasandoval22@gmail.com; 3 Laboratorio de Genética y Biología Molecular, Centro de Referencia para Lactobacilos (CERELA), San Miguel de Tucumán A4000, Argentina; lucila@cerela.org.ar

**Keywords:** leishmaniasis, *Enterococcus mundtii*, lactic acid bacteria, antileishmanial activity, cell-free supernatant, neglected tropical diseases, amphotericin B, cutaneous infection, therapeutic alternative

## Abstract

American tegumentary leishmaniasis (ATL) remains a therapeutic challenge due to the toxicity and limited effectiveness of conventional agents. This study evaluated *Enterococcus mundtii* CRL35, a food-grade lactic acid bacterium, as a dual-mechanism therapeutic platform against *Leishmania (L.) amazonensis*. Initial screening identified *E. mundtii* CRL35 as the most active strain among four enterococci tested, demonstrating high selectivity against promastigotes (SI = 95) and intracellular amastigotes (SI = 25). In a BALB/c murine model of cutaneous leishmaniasis, three treatment modalities were evaluated: topical application of cell-free supernatant (CFS-T), oral viable bacteria (LB-O), and their combination (CFS-T+LB-O). All three treatments achieved significant reductions in lesion parasite burden (approximately 2–3 orders of magnitude versus untreated controls), substantially exceeding the parasitological efficacy of amphotericin B under identical experimental conditions. CFS-T and CFS-T+LB-O additionally produced significant macroscopic lesion improvement. Importantly, no treatment was associated with adverse changes in organ indices or serum biochemical parameters. *E. mundtii* CRL35-based treatments promoted higher IgG2a/IgG1 antibody ratios compared to untreated controls and amphotericin B, generating an immune profile more favorable for intracellular parasite control. Taken together, these findings support the therapeutic potential of these modalities as safe, effective, and affordable alternatives for ATL, particularly in resource-limited endemic regions where toxicity and logistical barriers limit access to conventional therapy.

## 1. Introduction

American tegumentary leishmaniasis (ATL) remains a major public health concern, closely associated with poverty and affecting millions worldwide [1]. The disease causes cutaneous and mucocutaneous lesions that may lead to disfigurement, disability, and social stigma [2]. ATL exemplifies the intricate interdependence between human, animal, and environmental health, a relationship central to the One Health framework [3]. The absence of safe, effective, and accessible treatments underscores the urgent need for innovative therapeutic approaches.

ATL is caused by protozoa of the genus *Leishmania*, which are transmitted to humans by infected sandflies [4]. More than 20 pathogenic species have been identified that produce visceral, cutaneous, or mucocutaneous forms of the disease [1]. In Argentina, ATL predominates in the northern provinces, where *Leishmania (Viannia) braziliensis*, *Leishmania (Viannia) guyanensis*, and *Leishmania (Leishmania) amazonensis* are the main etiological agents [5,6,7,8,9]. Subtropical climates and socioeconomic vulnerability favor transmission, thereby hindering timely diagnosis and treatment.

Available treatments rely mainly on pentavalent antimonials, amphotericin B, and paromomycin, all of which present serious drawbacks. Meglumine antimoniate (Glucantime^®^), still used as first-line therapy in Argentina, causes pancreatitis, cardiotoxicity, and hepatotoxicity, necessitating close monitoring and frequently leading to treatment discontinuation [10,11]. Amphotericin B must be administered intravenously, often during hospitalization, due to risks of nephrotoxicity, hypokalemia, and myocarditis [12,13]. These limitations compromise patient adherence and significantly increase healthcare costs. Moreover, the emergence of resistant strains and coinfections (e.g., HIV/*Leishmania*) has exacerbated the therapeutic crisis [14]. The high frequency of therapeutic failures and adverse events underscores the need for alternative strategies that can improve adherence, reduce toxicity, and offer accessible administration options suitable for resource-limited endemic settings [10,12].

The current therapeutic pipeline for ATL remains limited. While natural products (polyphenols, alkaloids, and terpenoids) have shown promising activity, their development is often hindered by high costs, long timelines, and limited translational potential. Drug repurposing has accelerated development of safer treatments [10,15], but most repurposed drugs against *Leishmania* belong to antimicrobial classes originally developed against bacteria, fungi, or viruses, raising concerns about fueling antimicrobial resistance [10,13,16].

In this context, lactic acid bacteria (LAB), both as live microorganisms with direct immunomodulatory potential and as producers of bioactive metabolites with antimicrobial and antiparasitic properties, represent an alternative approach that has received limited attention in leishmaniasis research. LAB are ubiquitous microorganisms and natural inhabitants of the gastrointestinal tract in humans and animals [17]. Among LAB, *Enterococcus* species are particularly promising candidates given their robustness and well-documented probiotic properties [18]. Their tolerance to extreme pH, temperatures, and high salt concentrations enables *Enterococcus* strains to survive, compete, and adhere to host cells in diverse environments, features critical for probiotic applications [19]. Enterococci are extensively studied as probiotic candidates due to their capacity to produce antimicrobial compounds, including bacteriocins (also termed enterocins), which display broad-spectrum activity against Gram-positive foodborne pathogens such as *Listeria monocytogenes*, *Staphylococcus aureus*, and *Clostridium difficile* [18,20]. Recent evidence has expanded their potential therapeutic applications beyond bacterial pathogens, with enterocins demonstrating leishmanicidal effects at low micromolar concentrations [21], providing proof-of-concept for LAB-based anti-leishmanial strategies.

Beyond their direct antimicrobial activity, LAB exert strain-specific immunomodulatory effects through interaction with intestinal dendritic cells and macrophages, promoting the production of cytokines central to Th1-mediated cellular immunity against intracellular pathogens [22,23]. This capacity to shift the host immune balance toward a Th1 profile is particularly relevant in the context of *Leishmania* infection, where gut microbiota dysbiosis exacerbates disease pathogenesis through altered host immunity [24] and where a protective Th1 response is well established as essential for intracellular parasite control [25]. Despite these promising findings, the therapeutic potential of *Enterococcus* strains against ATL-causing *Leishmania* species has not been comprehensively evaluated using clinically relevant administration routes. Furthermore, the use of whole *Enterococcus* strains as orally delivered immunotherapeutic agents for cutaneous leishmaniasis remains largely unexplored. Organizations such as EFSA and the Food Standards Agency have permitted the use of specific *enterococcal* strains as food additives and supplements based on careful case-by-case safety assessments [26]. Several *Enterococcus* strains have been successfully commercialized and used safely for decades, including *E. faecium* SF68^®^ in probiotic preparations and *E. faecium* strains approved as feed additives [27,28]. These precedents not only support the feasibility of developing safe *Enterococcus*-based therapeutics through appropriate strain selection and safety validation but also encourage the exploration of new clinical applications beyond gut health [29]. In this context, drug combination therapy has been shown to enhance efficacy while reducing individual drug dosages and toxicity [30], whereas immunotherapy is gaining increasing attention as a complementary therapeutic strategy for neglected tropical diseases. Among the different immunotherapeutic approaches, probiotics have emerged as promising candidates due to their ability to modulate immune responses that favor pathogen control [31,32].

The aim of this study was to evaluate the antileishmanial potential of *Enterococcus* strains through a comprehensive preclinical assessment. We initially screened cell-free supernatants from four *Enterococcus* strains for antileishmanial activity in vitro against *L. (L.) amazonensis*. Subsequently, using a murine model of cutaneous leishmaniasis, we evaluated two therapeutic modalities: topical application of *E. mundtii* CRL35 cell-free supernatant to exploit potential direct antiparasitic effects of bacterial metabolites, and oral administration of viable *E. mundtii* CRL35 bacteria to characterize systemic antileishmanial control through an alternative delivery route. By integrating in vitro screening with comprehensive in vivo evaluation across both topical and oral administration routes, this study provides preclinical evidence supporting the development of *Enterococcus*-based approaches as safe, accessible, and effective alternatives for ATL treatment in endemic regions where current therapeutic options remain inadequate.

## 2. Materials and Methods

### 2.1. Parasites and Growth Conditions

*Leishmania (Leishmania) amazonensis* (MHOM/BR/73/M2269) was maintained by serial passages in tail-base lesions of BALB/c mice to preserve parasite virulence. Parasites were isolated from infected lesion borders by aseptic aspiration in sterile proline-balanced salt solution (PBSS) supplemented with antibiotics (100 U/mL of penicillin and 50 μg/mL of streptomycin). Aspirates were seeded in biphasic USMARU medium and maintained at 24 °C. Subcultures up to passage 4 post-isolation were used for promastigote screening assays, while subcultures up to passage 2 were used for all cell infection and in vivo studies to minimize virulence attenuation. After four days, cultures were transferred to RPMI 1640 medium (pH 7.5; Gibco, New York, NY, USA) supplemented with 10% heat-inactivated fetal bovine serum (FBS; Natocor, Córdoba, Argentina) and maintained at 24 °C. Exponential-phase promastigotes (3-day cultures) were used for screening assays, while stationary-phase metacyclic promastigotes (7-day cultures) were used for infection assays, as previously described [33,34].

### 2.2. Bacterial Strains and Preparation of Cell-Free Supernatants

Four *Enterococcus* strains isolated from fermented sources were used: *E. faecalis* IPE148, *E. faecalis* IPE89, *E. faecium* IPE114, (from our collection) and *E. mundtii* CRL35 (CERELA, Tucumán, Argentina). Strains were cultivated in Brain Heart Infusion (BHI) broth (Sigma-Aldrich, St. Louis, MO, USA) for 48 h at 37 °C with agitation, reaching final concentrations in the order of 10^8^ CFU/mL. Cell-free supernatants (CFS) were obtained by centrifugation at 14,500 rpm for 10 min and sterilized through 0.2-µm polyethersulfone membrane filters (Merck Millipore, Burlington, MA, USA), then stored at −20 °C until use.

### 2.3. In Vitro Antileishmanial Screening Against Promastigotes

Antileishmanial activity of cell-free supernatants (CFS) was evaluated against exponential-phase *Leishmania (Leishmania) amazonensis* promastigotes (1 × 10^6^ parasites/well) using eight two-fold serial dilutions in 96-well microplates. Plates were incubated at 24 °C for 48 h, after which parasite viability was determined by the WST-1 tetrazolium reduction assay by measuring absorbance at 570 nm, as previously described [33,35]. Amphotericin B (AmpB; Sigma-Aldrich, St. Louis, MO, USA) was included as a positive control under identical conditions. The percentage inhibition of parasite viability at each dilution was calculated and subjected to regression analysis using GraphPad Prism version 8.0 (GraphPad Software, San Diego, CA, USA) to determine the 50% inhibitory dilution (ID_50_). Standard errors of the mean (SEM) were derived from the regression analysis of three independent experiments, each performed in triplicate. Antileishmanial activity was subsequently expressed as arbitrary units per milliliter (AU/mL), adapting the conventional arbitrary-unit method based on the reciprocal of the ID_50_ [36,37]. AU/mL was calculated as AU/mL = (2^n^ × 1000)/p, where *n* represents the exponent of the ID_50_, and p is the volume (µL) of CFS assayed. Accordingly, higher AU/mL values indicate greater antileishmanial potency by reflecting the retention of inhibitory activity upon serial dilution.

### 2.4. Cytotoxicity Assay on RAW 264.7 Macrophages

Cytotoxic effects of CFS were evaluated in RAW 264.7 macrophages (ATCC TIB-71™) seeded at 2 × 10^5^ cells/well in 96-well microplates and incubated overnight for adhesion. Cells were then exposed for 48 h to eight 2-fold serial dilutions of each CFS, with AmpB (250 mg/mL in DMSO) as positive cytotoxicity control and untreated cells as negative control. Cell viability was determined by the WST-1 assay as described in Section 2.3. The percentage reduction in cell viability at each dilution was calculated and subjected to regression analysis using GraphPad Prism 8.0 to determine the 50% cytotoxic dilution (CD_50_). Standard errors of the mean (SEM) were derived from the regression analysis of three independent experiments, each performed in triplicate. Because antileishmanial activity was defined by the 50% inhibitory dilution (ID_50_), determined identically through regression analysis, higher values indicate greater potency; the selectivity index (SI) was calculated as SI = ID_50_/CD_50_. Accordingly, SI > 1 indicates greater selectivity toward parasites relative to host cells. This formulation preserves the biological interpretation of the selectivity index, whereby higher values consistently indicate greater antiparasitic potency relative to host-cell cytotoxicity.

### 2.5. Antileishmanial Activity Against Intracellular Amastigotes

The cell-free supernatant (CFS) exhibiting the highest activity against promastigotes was selected for subsequent evaluation against intracellular amastigotes using two complementary approaches: microscopic examination and quantitative rescue assay [33,38].

Microscopic Assessment. RAW 264.7 macrophages were seeded at 2 × 10^4^ cells/well on sterile round glass coverslips in 24-well plates (Nunc; Thermo Fisher Scientific, Waltham, MA, USA). After cell attachment, macrophages were infected with *L. (L.) amazonensis* metacyclic promastigotes at a parasite-to-macrophage ratio of 20:1 and incubated at 24 °C for 3 h. Following removal of non-internalized parasites by washing, infected macrophages were incubated for 48 h at 34 °C in 5% CO_2_ with increasing CFS concentrations. Cells were fixed with 4% paraformaldehyde (12 min), washed three times with PBS (pH 7.4), and stained using a rapid panoptic staining kit (Fast T15; Salttech, Buenos Aires, Argentina). Microscopic examination was performed on a Leica DM500 optical microscope (Mannheim, Germany) at 40× and 100× magnification, with images acquired using a Leica ICC50 HD camera in triplicate.

Quantitative Rescue Assay. RAW 264.7 macrophages were seeded at 2 × 10^4^ cells/well in flat-bottom 96-well plates (Nunc; Thermo Fisher Scientific) and subjected to identical infection and treatment protocols. Following CFS exposure, cells were lysed with 0.05% SDS in RPMI (30 s); released amastigotes were incubated in RPMI supplemented with 20% fetal bovine serum at 25 °C for 48 h to permit differentiation into promastigotes [38]. Promastigote viability was determined by WST-1 assay (Section 2.3), and IC_50_ and selectivity index (SI) were calculated as described in Section 2.4.

### 2.6. In Vivo Antileishmanial Evaluation in a Murine Model of Cutaneous Leishmaniasis

All animal experiments were conducted in accordance with NIH guidelines for the care and use of laboratory animals and were approved by the Institutional Animal Care and Use Committee of the Universidad Nacional de Salta (protocol number: 384-24). *Leishmania (L.) amazonensis* was selected as the model parasite because it is well-characterized for studying cutaneous American tegumentary leishmaniasis in BALB/c mice, exhibiting high susceptibility and reproducible pathology, and is also epidemiologically relevant as a causative agent of cutaneous leishmaniasis in Argentina, including in the province of Salta [39,40]. This strain is routinely maintained in our laboratory and has been extensively employed in prior preclinical studies of antileishmanial therapeutics [33,34,41,42,43]. Female BALB/c mice (8 weeks of age; *n* = 5 per group) were inoculated subcutaneously with 2 × 10^7^ *L*. (*L*.) *amazonensis* stationary-phase promastigotes at the base of the tail. This inoculum was selected based on established protocols in our laboratory that ensure consistent cutaneous infection establishment and measurable lesion development prior to treatment initiation, and is consistent with inocula employed in previous studies evaluating antileishmanial treatments in BALB/c mice [41,43,44]. At 60 days post-infection, animals were randomly assigned to five groups: (i) untreated infected control (ATL); (ii) AmpB, dissolved in PBS and administered i.p. at 4 mg/kg on alternating days, 3–4 doses per week for 6 weeks [45,46]; (iii) CFS-T, 100 µL of *E. mundtii* CRL35 CFS applied topically to the lesion once daily for 6 weeks [47,48]; (iv) LB-O, viable *E. mundtii* CRL35 (2.5 × 10^10^ CFU/mL) administered orally in 100 mL of drinking water available ad libitum, replaced every 48 h for 6 weeks [49,50]; and (v) CFS-T+LB-O, simultaneous topical CFS and oral live bacteria administered as described above. An additional group of healthy uninfected mice served as baseline reference.

Animals were monitored weekly for: (i) lesion area (mm^2^), quantified from digital photographs using ImageJ software v1.54g (National Institutes of Health, Bethesda, MD, USA) with a reference scale bar as reported [51]; (ii) body weight; and (iii) Macroscopic Clinical Severity Index (MCSI). Since similar-sized lesions presented distinct clinical characteristics depending on the treatment group, the MCSI was developed to capture qualitative differences related to lesion severity. The MCSI was adapted from [52,53] and consists of five parameters assessed by visual inspection of digital photographs: lesion size, wound borders, type of exudate, signs of inflammation, and signs of healing. Each parameter was scored according to predefined criteria (Table 1). For each experimental group, the mean score of each parameter was calculated across all animals, and these mean values were summed to obtain the Macroscopic Clinical Score (MCS). The MCS was then normalized to a percentage to generate the Macroscopic Clinical Severity Index (MCSI) according to the equation: $MCSI=\frac{\sum P_{i}}{P_{max}}\times 100$, where $\sum P_{i}$ is the sum of the mean scores for the five parameters and $P_{max}$ is the maximum possible score of the scale. Higher MCSI values indicate greater lesion severity. Based on the MCSI, lesions were classified into four severity categories: mild (1–25), moderate (26–50), severe (51–75), and extremely severe (76–100). At endpoint, animals were euthanized and spleen and liver were collected for parasitological, molecular, and toxicological analyses. Organ indices were calculated as: organ weight (mg)/body weight (g).

### 2.7. Parasite Burden Quantification by Limiting Dilution

Parasite burden in tail-base lesions was quantified by limiting dilution assay at the experimental endpoint, following the method of Lima et al. (1997) [54] with minor modifications. Skin tissue samples were aseptically excised and weighed to obtain standardized measurements. Tissues were initially washed in sterile phosphate-buffered saline solution (PBSS) supplemented with antimicrobial agents (100 U/mL penicillin and 50 mg/L streptomycin) to minimize bacterial contamination. Homogenization was performed in RPMI 1640 medium at a 1:10 tissue-to-medium ratio (*w*/*v*), followed by mechanical tissue disruption using a glass–Teflon homogenizer to liberate intracellular amastigotes. Serial two-fold dilutions of tissue homogenates were prepared in 96-well microplates containing complete RPMI 1640 culture medium. Plates were incubated at 24 °C in a humid atmosphere for 5 days, then examined at 40× magnification by inverted light microscopy to determine the highest dilution at which motile promastigotes were observed. The parasite load was calculated from the highest dilution showing active promastigotes and expressed as the total number of parasites per tissue sample.

### 2.8. DNA Extraction and Quantification of Parasite Burden in Spleen and Liver by qPCR

Genomic DNA was extracted from spleen and liver fragments using a commercial silica-column kit following the manufacturer’s protocol (Bioneer, Daejeon, Korea). DNA concentration and purity were assessed by NanoDrop spectrophotometry (Thermo Fisher Scientific, Waltham, MA, USA) and samples were normalized to 20 ng/µL prior to amplification. Quantitative PCR (qPCR) was performed using the species-specific primer pair LMi-amaF/MLR (LMi-amaF: 5′-AAAATGAGTGCAGAAACCC-3′; MLR: 5′-CGGCCCTATTTTACACCAACC-3′), targeting the *L. (L.) amazonensis* kinetoplast minicircle sequence [55]. Reactions (8 µL) contained 1 µL of DNA template, TB Green Premix Ex Taq II Mastermix (Takara Bio Europe, Saint-Germain-en-Laye, France), and 200 nM of each primer, and were run on a QuantStudio™ 5 Real-Time PCR System (Thermo Fisher Scientific) under the following conditions: 95 °C for 30 s, followed by 40 cycles of 95 °C for 5 s and 60 °C for 30 s, with a final melt-curve analysis. A standard curve was generated using serial dilutions of genomic DNA extracted from *L. (L.) amazonensis* parasites recovered from an untreated mouse lesion, whose parasite numbers had been previously determined by limiting dilution analysis [54,55]. For each sample, 40 ng of total DNA was used as the qPCR template. Parasite burden was calculated by interpolation from the standard curve and expressed as the number of parasites per 40 ng of total DNA. Group comparisons were performed using the Kruskal–Wallis test followed by Dunn’s multiple-comparison post hoc test.

### 2.9. Serum Biochemistry and Toxicological Parameters

Serum biochemical parameters were assessed at the experimental endpoint as indicators of systemic toxicity and hepatocellular damage [56]. Blood samples were collected by cardiac puncture under deep anesthesia, and serum was obtained by centrifugation at 3500 rpm for 10 min. Concentrations of urea, creatinine, aspartate aminotransferase (AST), alanine aminotransferase (ALT), and lactate dehydrogenase (LDH) were determined by an independent certified clinical laboratory (NEOLAB, Salta, Argentina) using routine automated biochemical methods.

### 2.10. Humoral Immune Response. Detection of Anti-Leishmania Antibodies by ELISA

*Leishmania*-specific IgG subclasses (IgG1 and IgG2a) and total IgG were quantified by indirect ELISA. Ninety-six-well microplates were coated overnight at 4 °C with 1 µg/well of total *L. (L.) amazonensis* antigen (LaAg) as previously reported [57,58] in carbonate–bicarbonate buffer (pH 9.6), then blocked with 5% skimmed milk in PBS-Tween 20 (PBST) for 1 h at 37 °C. Serum samples collected at baseline (pre-treatment) and at the experimental endpoint were incubated at a 1:100 dilution for 1 h at 37 °C. Biotin-conjugated anti-mouse IgG1 and anti-IgG2a antibodies (1:3500) were added and incubated for 1 h at 37 °C, followed by streptavidin–horseradish peroxidase conjugate for 1 h at 37 °C. Color was developed with O-phenylenediamine substrate (OPD; Sigma-Aldrich, St. Louis, MO, USA), and stopped with 0.5 N H_2_SO_4_, and absorbance was read at 495 nm using a microplate reader.

### 2.11. Statistical Analysis

Data are expressed as mean ± SEM. Comparisons among multiple groups were performed using one-way ANOVA followed by the Tukey post-hoc test. Statistical significance was set at *p* < 0.05. All analyses were performed using GraphPad Prism 8.0 (GraphPad Software, San Diego, CA, USA). All in vitro experiments were conducted in at least three independent experiments, each in triplicate.

## 3. Results

### 3.1. Screening of Enterococcus *sp.* Cell-Free Supernatants Against L. (L.) amazonensis Promastigotes

To assess the antileishmanial potential of *Enterococcus*-derived metabolites, cell-free supernatants (CFSs) from four *Enterococcus* strains were initially screened against *L. (L.) amazonensis* promastigotes. All four strains exhibited antileishmanial activity, though notable differences in potency and selectivity among strains were observed (Table 2).

*E. mundtii* CRL35 emerged as one of the most active strains, with an ID_50_ value of 1025 AU/mL, comparable to those of *E. faecium* IPE114 (1048 AU/mL) and *E. faecalis* IPE89 (1162 AU/mL), and exceeding *E. faecalis* IPE148 (705.7 AU/mL). However, the selection of *E. mundtii* CRL35 for further evaluation was determined not solely by antileishmanial potency, but by the integration of both antiparasitic and safety parameters. Cytotoxicity assays on RAW 264.7 macrophages revealed distinct safety profiles among strains. *E. faecalis* IPE148 displayed the lowest CD_50_ (8.54 AU/mL), indicating the lowest host cell toxicity among the strains evaluated, while *E. faecium* IPE114 and *E. faecalis* IPE89 showed the highest toxicity (CD_50_ = 20.21 and 20.2 AU/mL, respectively). *E. mundtii* CRL35 showed an intermediate CD_50_ of 10.81 AU/mL. As a reference, AmpB displayed an IC_50_ of 3.47 ± 0.61 µM and a CC_50_ of 8.24 ± 0.68 µM. When antileishmanial and cytotoxicity profiles were considered together through selectivity index (SI) calculation, a critical distinction emerged. Despite not displaying the lowest host cell toxicity, *E. mundtii* CRL35 presented the highest SI (95), reflecting the greatest differential between antileishmanial potency and host cell toxicity. *E. faecalis* IPE148 showed an intermediate SI (83), while *E. faecium* IPE114 and *E. faecalis* IPE89 presented the lowest selectivity (SI = 52 and 58, respectively). Notably, higher AU/mL values indicate activity retained at greater dilutions and therefore greater antiparasitic potency, while a higher SI reflects preferential activity against the parasite over the host macrophage line. Based on these integrated findings, we selected *E. mundtii* CRL35 as the most promising candidate among the strains evaluated. The SI value of 95 for *E. mundtii* CRL35 substantially exceeded that of AmpB (SI = 2.37), demonstrating markedly superior selectivity and supporting its selection for further characterization against intracellular amastigotes and subsequent in vivo assessment.

### 3.2. Antileishmanial Activity of E. mundtii CRL35 Supernatant Against Intracellular Amastigotes

Following the initial screening, the antileishmanial potential of *E. mundtii* CRL35 CFS was further characterized against intracellular amastigotes to assess activity across parasite developmental stages relevant to human infection. The intracellular amastigote stage of *L. (L.) amazonensis*, represents the clinically relevant form of the parasite within host macrophages and is generally more resistant to chemotherapeutic agents than promastigotes. Using the indirect rescue assay, CFS inhibited amastigote viability with an ID_50_ of 270.2 AU/mL (Table 3).

**Figure 1:**
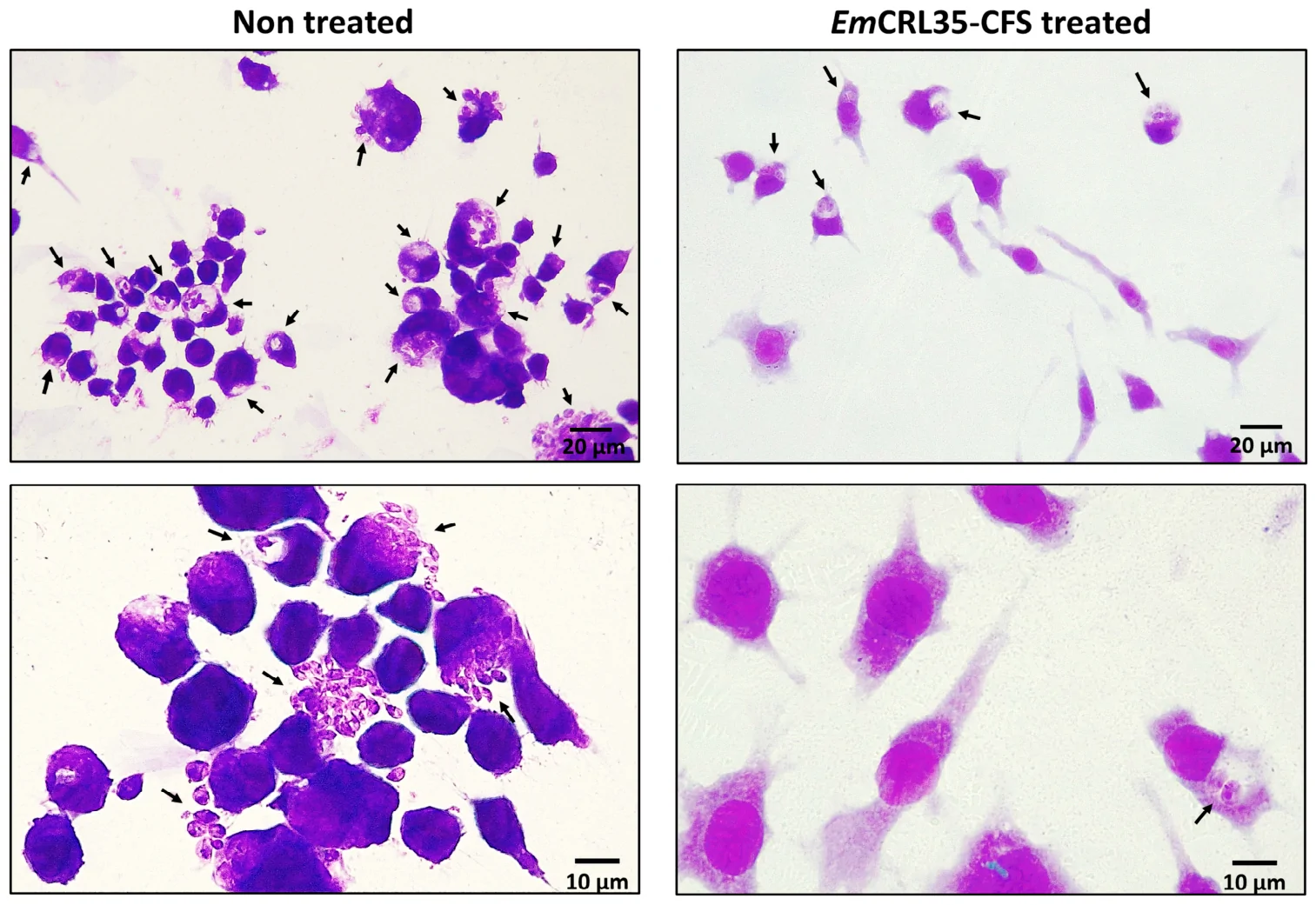
Representative antileishmanial activity of *E. mundtii* CRL35 cell-free supernatant (*Em*CRL35-CFS) against intracellular *L. (L.) amazonensis* amastigotes. Optical microscopy images of RAW 264.7 macrophages infected with *L.* (*L*.) *amazonensis* amastigotes displayed at 40× (upper panels) and 100× magnification (lower panels). Left panels show untreated infected macrophages with dense parasite burden; right panels depict macrophages treated with 2 × ID_50_ (540 AU/mL) of CFS, demonstrating marked parasite reduction. Black arrows indicate intracellular amastigotes. Images are representative of three independent experiments, each performed in triplicate.

Using the CD_50_ determined in Section 3.1 (10.8 AU/mL), the resulting SI for the amastigote stage was 25, notably lower than that observed against promastigotes (SI = 95). Despite this reduction, the SI remained well above 1, indicating a favorable selectivity profile at the intracellular stage. Qualitative microscopic analysis of the direct assay corroborated these findings at the cellular level (Figure 1). Untreated infected macrophages exhibited high intracellular parasite burden, with numerous amastigotes visible within host cells (Figure 1, left panels). In contrast, macrophages treated with 2 × ID_50_ (540 AU/mL) of CFS displayed a marked reduction in intracellular parasite load, with the majority of cells presenting few or no detectable amastigotes (Figure 1, right panels). These microscopic observations align with the quantitative viability data, confirming that the reduced parasite burden reflects genuine loss of amastigote viability rather than mere inhibition of proliferation. Collectively, these results demonstrate that *E. mundtii* CRL35 CFS retains significant antileishmanial activity against the intracellular amastigote stage, extending the findings observed against promastigotes and providing a robust biological rationale for its subsequent evaluation in an in vivo model of cutaneous leishmaniasis.

### 3.3. In Vivo Effects of E. mundtii CRL35-Based Treatments on Lesion Severity and Parasite Burden

**Figure 2:**
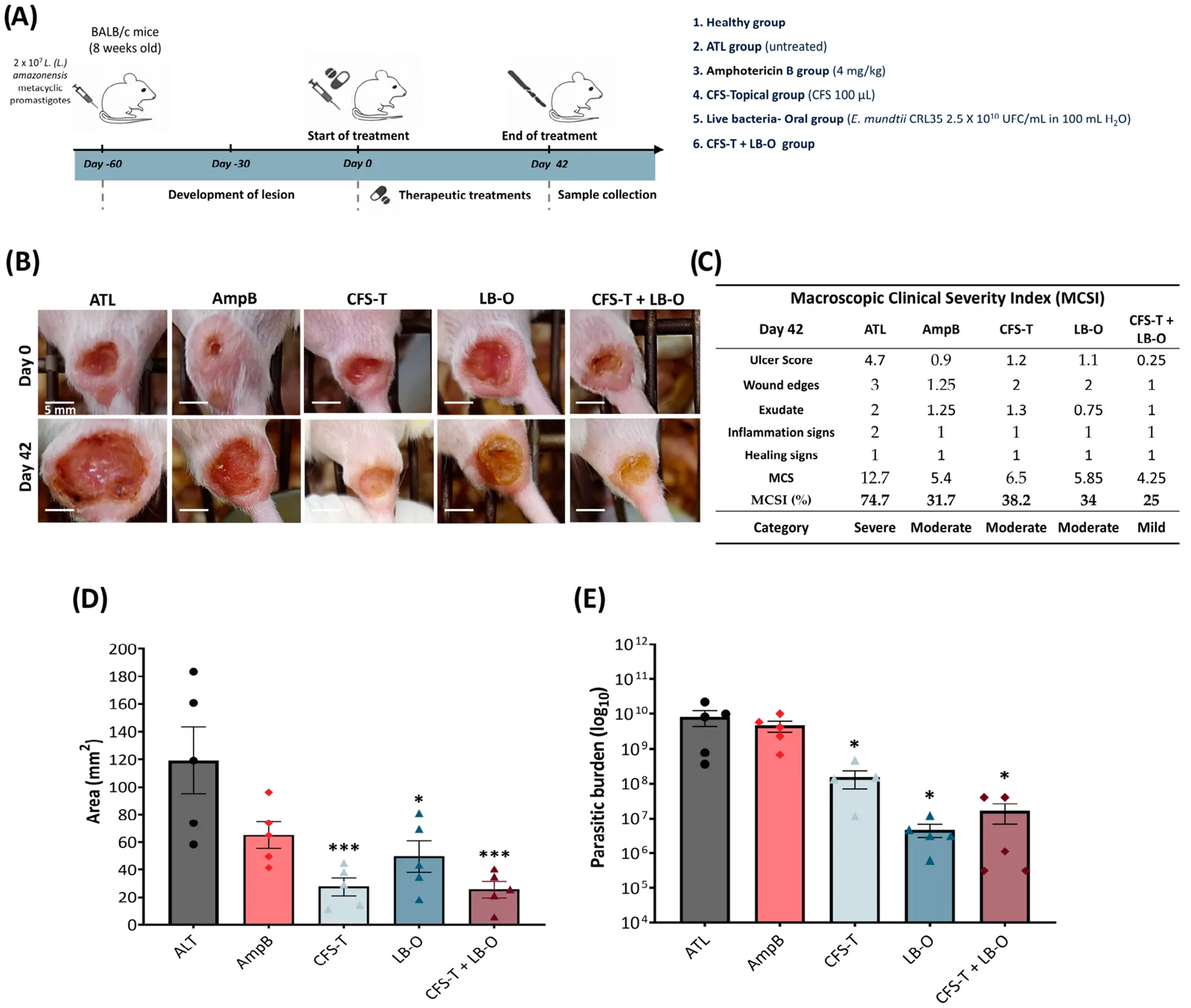
In vivo therapeutic effects of *E. mundtii* CRL35 treatments on lesion progression and parasite control. (**A**) Experimental timeline showing infection, lesion development monitoring, and 6-week treatment period. (**B**) Representative macroscopic images of cutaneous lesions comparing day 0 and day 42 post-treatment outcomes across experimental groups. Scale bars = 5 mm. (**C**) Macroscopic Clinical Severity Index (MCSI) quantification at day 42, with higher values reflecting increased lesion severity. (**D**) Lesion area (mm^2^) measurement and (**E**) parasite burden (log_10_ parasites/mL) quantified in lesions at experimental endpoint. Experimental groups: ATL (untreated infected control), AmpB (amphotericin B, 4 mg/kg i.p.), CFS-T (*E. mundtii* CRL35 CFS applied topically to lesions), LB-O (viable *E. mundtii* CRL35 administered orally in drinking water), and CFS-T+LB-O (simultaneous topical CFS and oral live bacteria). Data are presented as mean ± SEM with individual values. Statistical comparisons vs. ATL control: * *p* < 0.05, *** *p* < 0.001.

Building on the in vitro activity observed against both promastigotes and intracellular amastigotes, the therapeutic potential of *E. mundtii* CRL35 was evaluated in a murine model of cutaneous leishmaniasis using three treatment modalities: topical application of CFS (CFS-T), oral administration of viable bacteria (LB-O), and their combination (CFS-T + LB-O) (Figure 2A). AmpB served as the reference drug. Macroscopic examination of cutaneous lesions at day 0 and day 42 (Figure 2B) revealed visible differences in lesion appearance across treatment groups. At day 42, lesions in the untreated ATL control remained large, open, and with evident inflammatory features. Animals treated with AmpB showed partial improvement, with lesions still presenting an open appearance but with some reduction in size. In contrast, both CFS-T and LB-O displayed more advanced healing, with lesions showing crust formation and reduced inflammatory signs. The most notable macroscopic improvement was observed in the CFS-T + LB-O group, which exhibited markedly smaller lesions with prominent crust formation and signs of re-epithelialization, suggesting superior healing progression compared to all other groups.

To provide a more comprehensive and semi-quantitative evaluation of these macroscopic differences, a Macroscopic Clinical Severity Index (MCSI) was applied, integrating five key parameters: lesion size, wound edges, exudate type, inflammation signs, and healing, into a single composite score (Figure 2C). At day 42, the MCSI confirmed that all treatments reduced lesion severity relative to the untreated ATL group, which exhibited the highest index (74.7%) corresponding to severe classification. AmpB (31.7%), CFS-T (38.2%), and LB-O (34%) reduced the MCSI to moderate classification, indicating partial improvement. Notably, the combined treatment (CFS-T + LB-O) produced the greatest therapeutic effect, yielding the lowest MCSI (25%) and improving lesions to the mild category, making it the only treatment to achieve this level of clinical improvement. Importantly, the MCSI demonstrated that lesion severity was not determined solely by ulcer size. Although some treatment groups presented lesions of comparable dimensions, differences in wound edge morphology, inflammatory signs, and healing characteristics contributed to distinct composite scores. This finding highlights the value of the MCSI as a multidimensional clinical assessment, integrating both lesion size and qualitative features of tissue damage and repair. No evidence of severe secondary infection, such as purulent exudate or friable tissue, was observed in any treated group.

Consistent with the macroscopic observations and the MCSI evaluation, quantitative analysis of lesion area at day 42 confirmed that CFS-T and the combined treatment (CFS-T + LB-O) produced the greatest reductions in lesion area compared with the untreated ATL control (both *p* < 0.001), while LB-O also achieved a statistically significant reduction (*p* < 0.05 vs. ATL) (Figure 2D). In contrast, AmpB did not produce a statistically significant reduction in lesion area compared with ATL, indicating that both topical application of CFS-T alone and its combination with LB-O were more effective than conventional AmpB therapy at reducing lesion size.

In line with the macroscopic improvements described above, quantification of parasite burden by the limiting dilution assay at day 42 (Figure 2E) revealed a significant decrease in parasite load in all three *E. mundtii* CRL35-based treatment groups compared with the untreated ATL control (*p* < 0.05 for CFS-T, LB-O, and CFS-T + LB-O). Specifically, parasite burden was reduced by approximately two orders of magnitude following CFS-T treatment and by nearly three orders of magnitude in the LB-O and CFS-T + LB-O groups. In contrast, AmpB-treated animals showed parasite burdens comparable to those of the untreated ATL control, with no statistically significant reduction detected. Taken together, these results demonstrate that all three *E. mundtii* CRL35-based treatments achieved significant reductions in lesion parasite burden, an effect not observed with AmpB under the same experimental conditions. Furthermore, CFS-T and CFS-T + LB-O produced superior macroscopic outcomes compared to AmpB, achieving advanced lesion closure at day 42. These findings provide robust in vivo evidence supporting *E. mundtii* CRL35-based treatments as promising alternatives to conventional antileishmanial therapy and prompted further evaluation of systemic parameters.

**Figure 3:**
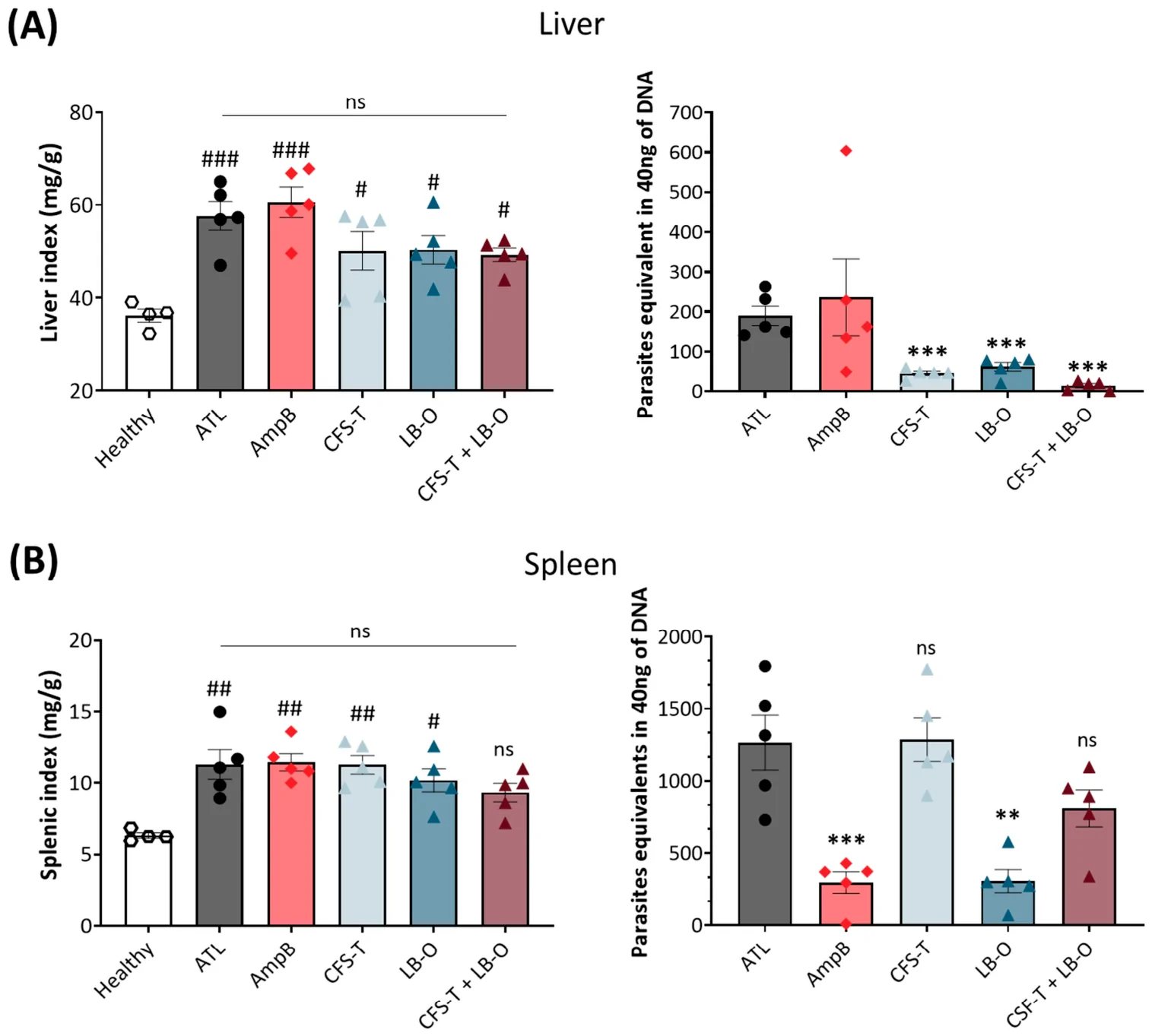
Organ indices and systemic parasite burden. (**A**) Hepatic index (left panel) and (**B**) splenic index (left panel) expressed as organ weight (mg) per body weight (g) ((**A**), right panel) Liver parasite burden and ((**B**), right panel) spleen parasite burden quantified by qPCR and expressed as number of parasites per 40 ng of DNA. Experimental groups: ATL (untreated infected control), AmpB (amphotericin B, 4 mg/kg i.p.), CFS-T (*E. mundtii* CRL35 cell-free supernatant applied topically to lesions), LB-O (viable *E. mundtii* CRL35 administered orally in drinking water), and CFS-T+LB-O (simultaneous topical CFS and oral live bacteria). Data are presented as individual values with mean ± SEM. Statistical comparisons: # *p* < 0.05, ## *p* < 0.01, and ### *p* < 0.001 vs. healthy; ** *p* < 0.01, and *** *p* < 0.001 vs. ATL; ns, not significantly different.

To assess whether the treatments influenced systemic disease progression and parasite dissemination beyond the primary lesion site, hepatic and splenic indices as well as organ-specific parasite burdens were evaluated at the experimental endpoint. All infected groups exhibited higher hepatic indices than healthy controls (Figure 3A, left panel), consistent with the hepatomegaly associated with infection. Among infected groups, the untreated ATL control and AmpB-treated mice showed the greatest hepatic enlargement with indices significantly higher than those of healthy control (*p* < 0.001). In contrast, the *E. mundtii* CRL35-based treatments (CFS-T, LB-O, and CFS-T+LB-O) displayed hepatic indices numerically lower than the untreated ATL group, although without statistically significant differences between treatment groups. All three *E. mundtii* CRL35 treatments remained elevated compared with healthy animals (*p* < 0.05), yet this elevation appeared more modest than that observed in untreated infection, which may suggest partial attenuation of infection-associated liver enlargement (Figure 3A, left panel). A similar pattern was observed for splenic indices, which were significantly increased in all infected groups relative to healthy controls reflecting infection-induced splenomegaly. However, LB-O-treated animals showed splenic indices approaching those of healthy control (*p* < 0.05 vs. healthy), whereas the combined treatment (CFS-T+LB-O) did not differ significantly from healthy animals, indicating the greatest reduction in splenic enlargement among the treatments evaluated (Figure 3B, left panel).

Parasite dissemination to the liver and spleen was quantified by qPCR at the experimental endpoint (Figure 3A,B, right panels). In the liver, all *E. mundtii* CRL35-based treatments (CFS-T, LB-O, and CFS-T + LB-O) significantly reduced parasite burden compared with both the untreated ATL control and AmpB-treated groups (*p* < 0.001), resulting in the lowest hepatic parasite loads among all infected groups (Figure 3A, right panel). In contrast, splenic parasite burden showed marked inter-individual variability, which limited statistical comparisons between treatment groups. Despite this variability, AmpB and LB-O significantly reduced splenic parasite loads relative to the ATL control (*p* < 0.001 and *p* < 0.01, respectively), whereas CFS-T and CFS-T + LB-O did not differ significantly from ATL (Figure 3B, right panel).

Overall, the *E. mundtii* CRL35-based formulations were associated with lower hepatic indices than the untreated ATL group, whereas AmpB produced the greatest liver enlargement among all infected groups. Moreover, all three formulations significantly reduced hepatic parasite burden compared with AmpB, highlighting their improved capacity to limit liver infection. In the spleen, although parasite burden showed substantial inter-individual variability, LB-O achieved reductions comparable to those observed with AmpB despite being administered orally, while the combined treatment (CFS-T + LB-O) provided similar parasitological control without increasing splenic enlargement. Collectively, these findings indicate that the *E. mundtii* CRL35-based formulations effectively limited parasite dissemination while mitigating infection-associated organ alterations, supporting their further evaluation as alternative therapeutic strategies for ATL.

### 3.4. Systemic Humoral Immune Response During Treatments

**Figure 4:**
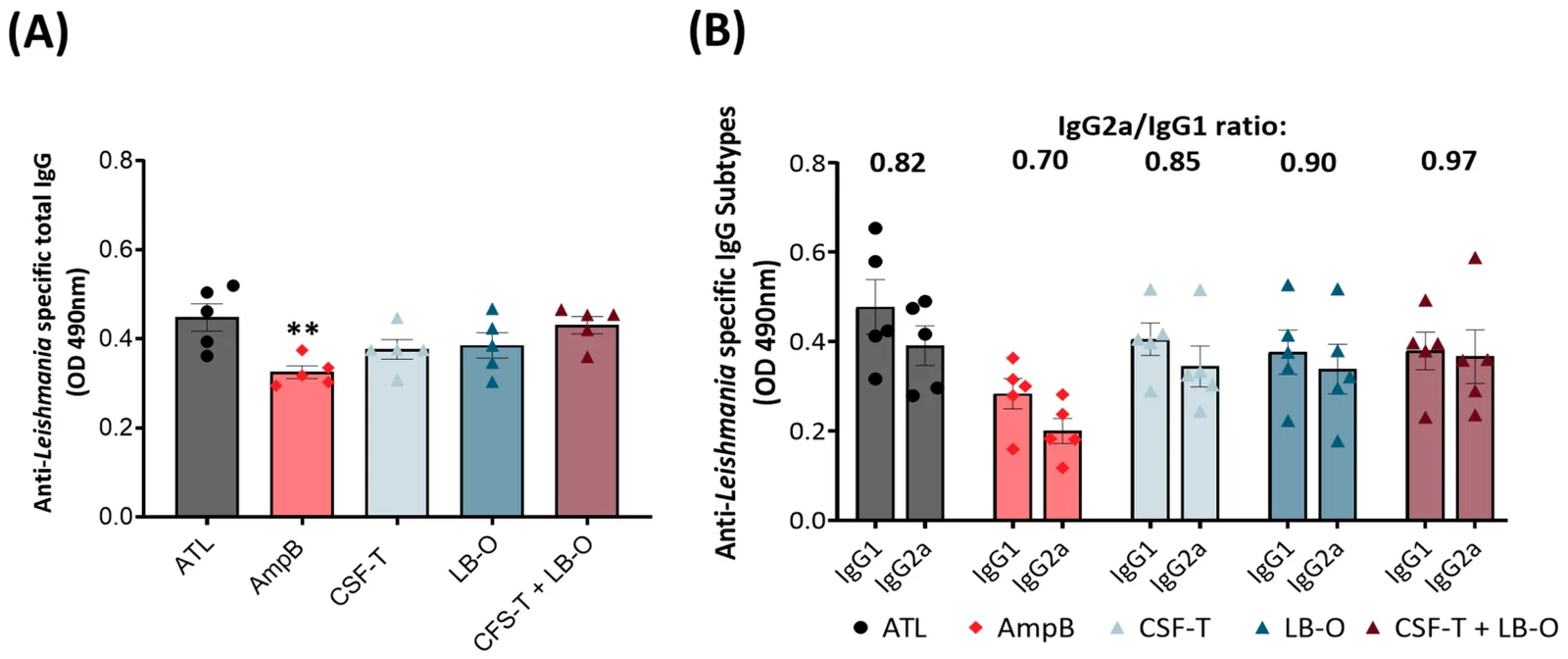
Systemic humoral immune response during treatments. (**A**) Total IgG levels at the experimental endpoint. (**B**) IgG1 and IgG2a levels at the experimental endpoint, with IgG2a/IgG1 subclass ratios displayed as insets above each treatment group. Experimental groups: healthy (uninfected control), ATL (untreated infected control), AmpB (amphotericin B, 4 mg/kg i.p.), CFS-T (*E. mundtii* CRL35 cell-free supernatant applied topically to lesions), LB-O (viable *E. mundtii* CRL35 administered orally in drinking water), and CFS-T+LB-O (simultaneous topical CFS and oral live bacteria). Data are presented as individual values with mean ± SEM. Statistical significance vs. ATL control: ** *p* < 0.01.

To evaluate whether *E. mundtii* CRL35-based treatments influenced adaptive immune responses, anti-*Leishmania* specific antibody levels were quantified at baseline and at the experimental endpoint. At the experimental endpoint, total IgG levels were significantly lower in AmpB-treated mice than in the ATL control group (*p* < 0.01) (Figure 4A). In contrast, CFS-T, LB-O, and CFS-T+LB-O, maintained total IgG levels comparable to those of the ATL controls (Figure 4A). Analysis of IgG subclass levels revealed a predominance of IgG1 over IgG2a across all infected groups, consistent with the Th2-biased immune response characteristic of BALB/c mice (Figure 4B). However, the IgG2a/IgG1 ratio varied among treatment groups. The ATL control exhibited a ratio of 0.82, whereas AmpB-treated animals showed the lowestr ratio of 0.70, indicating a relative reduction in IgG2a production. By contrast, all *E. mundtii* CRL35-based treatments exhibited higher IgG2a/IgG1 ratios, with CFS-T reaching 0.85, LB-O 0.90, and the combined treatment (CFS-T+LB-O) showing the highest ratio (0.97) (Figure 4B).

Longitudinal analysis of IgG subclass responses showed that the differences in IgG2a/IgG1 ratios were primarily driven by changes in IgG1 rather than IgG2a levels. Indeed, IgG2a remained relatively stable throughout the study, with only minor variations in mean optical density (ΔOD) across groups: 0.03 (ATL), −0.05 (AmpB), 0.04 (CFS-T), 0.09 (LB-O), and −0.07 (CFS-T + LB-O). In contrast, IgG1 levels increased over time in all groups, although the magnitude of this increase differed according to treatment. The greatest increases were observed in ATL (ΔOD = 0.223) and LB-O (ΔOD = 0.218), followed by CFS-T (ΔOD = 0.191) and the combined treatment CFS-T + LB-O (ΔOD = 0.165), whereas AmpB showed the smallest increase (ΔOD = 0.097). These findings indicate that the higher IgG2a/IgG1 ratios observed in the *E. mundtii* CRL35-based formulations were mainly attributable to a more restrained increase in IgG1, rather than to enhanced IgG2a production.

### 3.5. Assessment of Liver and Kidney Function During Treatment

**Figure 5:**
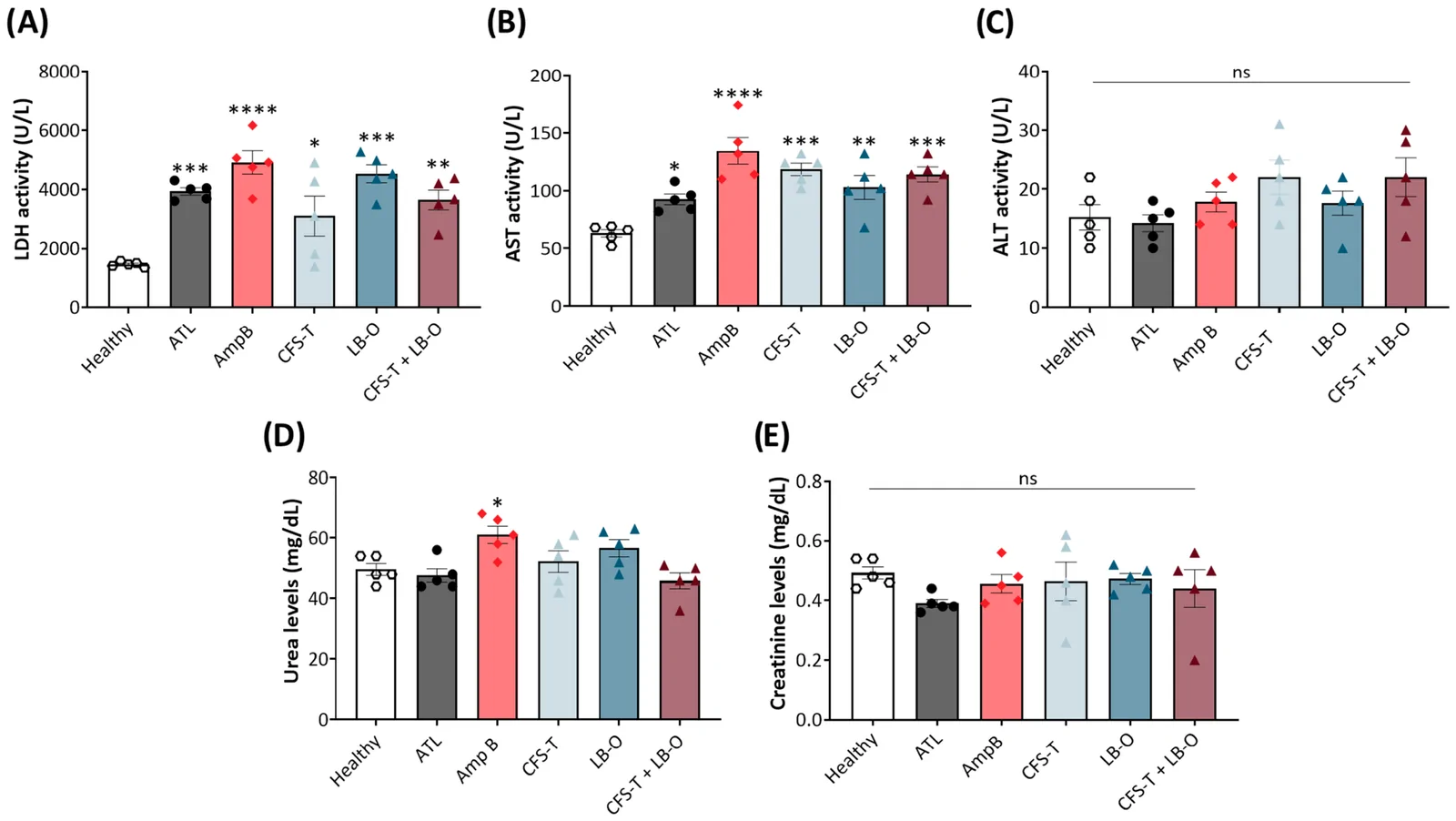
Serum biochemical parameters. (**A**) Lactate dehydrogenase (LDH) as a marker of tissue damage, (**B**) Aspartate aminotransferase (AST), and (**C**) Alanine aminotransferase (ALT) as indicators of hepatic function. (**D**) Urea levels and (**E**) Creatinine levels as markers of renal function, all quantified at the experimental endpoint. Experimental groups: healthy (uninfected control), ATL (untreated infected control), AmpB (amphotericin B, 4 mg/kg i.p.), CFS-T (*E. mundtii* CRL35 cell-free supernatant applied topically to lesions), LB-O (viable *E. mundtii* CRL35 administered orally in drinking water), and CFS-T+LB-O (simultaneous topical CFS and oral live bacteria). Data are presented as individual values with mean ± SEM. Statistical significance vs. healthy control: * *p* < 0.05, ** *p* < 0.01, *** *p* < 0.001, and **** *p* < 0.0001.

To comprehensively evaluate the safety profile of *E. mundtii* CRL35-based treatments, serum biochemical markers of tissue damage, as well as hepatic and renal function were quantified at the experimental endpoint (Figure 5). Compared with healthy controls, ATL-infected mice exhibited significantly elevated LDH levels (*p* < 0.001) (Figure 5A). This increase was further accentuated in AmpB-treated mice, which showed significantly higher LDH concentrations than both healthy and ATL-infected animals (*p* < 0.0001), suggesting greater systemic toxicity associated with conventional therapy. By contrast, *E. mundtii* CRL35-based treatments displayed variable reductions in LDH levels compared to healthy controls. CFS-T showed the most modest elevation (*p* < 0.05 vs. healthy), while LB-O and CFS-T + LB-O remained significantly elevated above healthy levels (*p* < 0.001 and *p* < 0.01, respectively) but displayed lower mean values than both ATL and AmpB-treated animals (Figure 5A). AST levels were moderately increased in ATL-infected mice compared with healthy controls (*p* < 0.05) (Figure 5B). This increase was markedly greater in AmpB-treated animals, which displayed significantly higher AST levels than healthy mice (*p* < 0.0001). By contrast, all *E. mundtii* CRL35-based treatments, namely CFS-T, LB-O, and CFS-T+LB-O, displayed AST levels elevated above healthy controls (*p* < 0.001, *p* < 0.01, and *p* < 0.001, respectively), though numerically lower than AmpB-treated animals (Figure 5B). ALT levels remained within the range observed in healthy controls and did not differ significantly among groups (Figure 5C). Overall, these biochemical findings indicate that conventional AmpB treatment was associated with greater hepatic injury than infection alone, whereas the *E. mundtii* CRL35-based formulations maintained liver enzyme levels comparable to those of untreated infected animals. Regarding renal function, urea levels were significantly elevated in AmpB-treated mice compared with both healthy controls and the ATL-infected group (*p* < 0.05) (Figure 5D). In contrast *E. mundtii* CRL35-based treatments (CFS-T, LB-O, and CFS-T+LB-O) maintained urea concentrations comparable to those of healthy animals, with no significant differences detected. Creatinine levels remained stable across all experimental groups, with no statistically significant differences detected (Figure 5E).

Overall, the biochemical profiles associated with the *E. mundtii* CRL35-based formulations remained consistently closer to those of healthy controls than to those of AmpB-treated animals. Although infection itself was associated with increased LDH and AST levels, these alterations were markedly accentuated by AmpB treatment, whereas the *E. mundtii* CRL35-based formulations maintained hepatic and renal biomarkers within the range observed in untreated infected animals, without evidence of additional organ toxicity.

## 4. Discussion

This study provides the first preclinical evidence that *Enterococcus mundtii* CRL35 represents a dual-mechanism therapeutic platform against American tegumentary leishmaniasis, acting both as a direct antiparasitic agent when applied topically and as an orally administered viable bacteria with systemic antiparasitic effects consistent with immunomodulatory mechanisms. The demonstration that a food-grade bacterium achieves parasitological and safety outcomes comparable or superior to amphotericin B, one of the main reference drugs in conventional leishmaniasis therapy, challenges the reliance on toxic small-molecule treatments and establishes a proof-of-principle for viable bacteria-based therapeutics in neglected tropical diseases.

### 4.1. Antileishmanial Efficacy: A Dual-Route Therapeutic Approach

The use of bacterial metabolites and cell-free supernatants to control protozoan infections remains considerably less explored than their application against bacterial and fungal pathogens. Nevertheless, emerging evidence demonstrates that probiotic bacteria-derived products can display antiparasitic activity. Cell-free supernatants from *Lactobacillus acidophilus* and *L. reuteri* reduced *Cryptosporidium parvum* oocyst viability by 40–80% in vitro [59,60], while *L. rhamnosus* supernatant exhibited anticoccidial activity against *Eimeria tenella*, reducing oocyst shedding in experimentally infected chickens [61]. More recent investigations have focused on entomopathogenic bacteria, particularly *Xenorhabdus* and *Photorhabdus* species, which produce diverse bioactive secondary metabolites. Cell-free supernatants from several *Xenorhabdus* species (*X. budapestensis*, *X. cabanillasii*, *X. indica*, *X. innexi*, and *X. miraniensis*) displayed substantial antiprotozoal activity, including activity against *Leishmania tropica* and *Trypanosoma cruzi*, with >80% inhibition reported [62]. Additionally, *X. budapestensis* and *X. szentirmaii* supernatants were active against *L. (L.) amazonensis* promastigotes [63]. Notably, these studies evaluated bacterial metabolites exclusively against the extracellular promastigote stage; activity against the clinically relevant intracellular amastigote stage remains largely unexplored.

In this context, the antileishmanial activity of *E. mundtii* CRL35 is distinguished by its selective toxicity against both *L. (L.) amazonensis* promastigotes and intracellular amastigotes in vitro, confirming that bacterial metabolites retain activity across parasite developmental stages relevant to human infection. Genomic analysis of *E. mundtii* CRL35 has identified genes encoding antimicrobial peptides, lipases, esterases, phospholipases, and oligopeptide transport systems [64], suggesting that the antileishmanial activity of the CFS likely reflects the combined action of multiple bioactive metabolites. The selectivity profile of *E. mundtii* CRL35 CFS compares favorably with natural antileishmanial compounds evaluated against *L. (L.) amazonensis*: (−)-epigallocatechin gallate (EGCG), the most abundant flavonoid in green tea, exhibited an SI of 129.4 against intracellular amastigotes through mitochondrial disruption [65], closely approaching that of CRL35 CFS against promastigotes (SI = 95), while CRL35 CFS against amastigotes (SI = 25) outperformed beauvericin, a cyclic depsipeptide ionophore acting through membrane ion permeabilization (SI = 22–30) [66]. The convergence of distinct mechanisms of membrane disruption across these natural compounds supports the concept that *Leishmania* parasite membranes represent a particularly vulnerable therapeutic target. In this way, membrane-active bacteriocins have demonstrated in vitro antiparasitic effects, most notably enterocin AS-48 against *Leishmania* spp. through plasma membrane permeabilization and mitochondrial dysfunction [21]. The present findings could extend this principle to *E. mundtii* CRL35, which produces enterocin CRL35, a class IIa bacteriocin with demonstrated membrane-permeabilizing activity [67,68,69,70], although the relative contribution of enterocin CRL35 versus other CFS components to the observed antileishmanial activity remains to be determined. In addition, *E. mundtii* CRL35 metabolites have also demonstrated antiviral activity against herpes simplex virus [71] and antifungal activity against *Candida albicans* [72], further supporting the hypothesis that CFS components target conserved membrane structures across phylogenetically diverse pathogens. Beyond the direct antiparasitic activity of the CFS, oral administration of viable *E. mundtii* CRL35 represented a distinct therapeutic approach whose antiparasitic effects were evaluated in parallel. Oral administration of probiotic enterococci modulates systemic immune responses through multiple pathways [22,73,74]. The systemic antiparasitic effects observed with LB-O, in the absence of direct tissue delivery of bioactive compounds, provide the first experimental evidence that these immunomodulatory properties translate into measurable antiparasitic outcomes in a cutaneous leishmaniasis model, though the specific mechanisms remain to be fully elucidated.

With respect to local antiparasitic activity, topical application of antimicrobial natural products against cutaneous leishmaniasis has gained increasing attention as a strategy to maximize local bioavailability while minimizing systemic toxicity. Among natural compounds evaluated topically in murine models of LTA, EGCG achieved significant reductions in lesion size and parasite burden in *L. (L.) amazonensis*-infected mice, with efficacy comparable to glucantime [75]. Additionally, triphenylmethane derivatives eliminated parasite burdens at the lesion site in *L. (L.) amazonensis*-infected BALB/c mice [76]. For *L. (V.) braziliensis*, which is also causative of LTA in endemic regions, piperine, quercetin, and curcumin demonstrated promising topical antileishmanial activity [77]. The present study extends this line of investigation to *Enterococcus* sp. metabolites, demonstrating that topical CFS application achieves comparable parasitological outcomes through a mechanism yet to be fully characterized. The limited systemic effect of CFS-T, reflected in higher splenic parasite loads relative to AmpB, is consistent with the pharmacokinetic profile of topically applied formulations where high local concentrations are achieved with minimal systemic absorption [47]. Notably, while systemically administered AmpB did not achieve significant lesion parasite reduction, topical CFS application produced significant local parasitological improvement, highlighting the potential advantage of targeted local delivery over conventional systemic therapy for the cutaneous form of the disease. While CFS-T exerted its effects primarily at the lesion site, the parasitological outcomes achieved by LB-O represent the most conceptually novel finding of this study. Since viable bacteria administered orally would not be expected to reach cutaneous lesions or visceral organs at concentrations sufficient for direct antimicrobial activity, the observed local and systemic parasite control is consistent with immunomodulatory mechanisms as the primary driver of LB-O efficacy. Recent evidence demonstrates that *L. (L.) amazonensis* inoculum size directly influences lesion progression and parasite dissemination kinetics [78]. Against this background, the parasitological outcomes observed with LB-O are particularly noteworthy: despite the use of a high inoculum (2 × 10^7^ promastigotes), LB-O significantly reduced parasite burden at both the lesion site and in visceral organs, indicating apparent limitation of infection-associated parasite dissemination. This effect is consistent with a systemic mechanism of action and supports the hypothesis that oral LB-O exerts antiparasitic efficacy through immunomodulation rather than direct antimicrobial activity. In this regard, oral probiotic bacteria have been shown to modulate systemic and mucosal immunity through Th1 polarization, macrophage activation, and increased production of IFN-γ and TNF-α, cytokines critical for intracellular parasite killing, which may underlie the antiparasitic effects observed here [22,24,79]. The absence of significant lesion area reduction in LB-O, despite significant reductions in lesion parasite burden, is consistent with the kinetics of *L. (L.) amazonensis* infection, where lesion closure lags parasite clearance due to the time required for tissue remodeling and inflammation resolution [43]. Extended treatment courses would clarify whether immune-mediated parasite control ultimately translates into macroscopic healing comparable to direct antiparasitic interventions. Given that available evidence have primarily explored probiotic bacteria or their derived metabolites as individual approaches, the combination of topical CFS-T and oral LB-O represents a novel strategy that may integrate the local anti-*Leishmania* effects of bacterial-derived metabolites with the potential systemic immunomodulatory effects of viable probiotic bacteria. The combination of both modalities in the CFS-T+LB-O group produced the greatest reduction in lesion area and significant hepatic parasite control superior to AmpB, and was the only group to reach mild severity in the MCSI. However, lesion parasite burden reductions were comparable to those of CFS-T and LB-O individually, suggesting that the two modalities may not produce additive parasitological effects at the lesion level within the 6-week period evaluated. Optimization of dosing regimens and treatment duration may be required to capture potential synergistic benefits, as demonstrated for combination strategies in leishmaniasis models [30].

The humoral immune profiles induced by *E. mundtii* CRL35-based treatments revealed a critical mechanistic distinction from conventional therapy. While all infected groups maintained predominant IgG1 responses consistent with the Th2-biased phenotype of BALB/c mice, quantitative analysis of IgG subclass ratios demonstrated a progressive shift toward higher IgG2a/IgG1 ratios in *E. mundtii* CRL35-treated animals. Specifically, the combined treatment CFS-T+LB-O achieved the highest IgG2a/IgG1 ratio, substantially exceeding both the untreated ATL control and particularly AmpB-treated animals. This shift was driven by attenuated IgG1 production in *E. mundtii* treatments compared to ATL, while IgG2a production remained stable across all groups, resulting in a more balanced subclass profile. Elevated IgG2a/IgG1 ratios are associated with enhanced Th1-biased responses necessary for intracellular pathogen control in leishmaniasis, generating a protective immune profile more favorable than the Th2-skewed response characteristic of progressive untreated infection [80,81]. In contrast, AmpB-treated mice exhibited suppressed total IgG, IgG1, and IgG2a levels, coupled with a reduced IgG2a/IgG1 ratio, suggesting potential immunomodulatory effects that may limit adaptive immune engagement during treatment. This distinction underscores a fundamental advantage of *E. mundtii* CRL35-based treatments: they sustain or enhance adaptive immunity while simultaneously promoting a more Th1-favorable antibody profile, supporting rather than compromising host immune competence during treatment.

### 4.2. Safety and Tolerability: A Marked Contrast to Amphotericin B

The toxicity of AmpB, evidenced by significantly elevated LDH, AST, and urea is consistent with its well-documented systemic toxicity profile [12,13]. In contrast, all three *E. mundtii* CRL35-based treatments maintained biochemical profiles comparable to the untreated ATL group, demonstrating that therapeutic efficacy was achieved without exacerbating infection-associated organ involvement. These safety outcomes are consistent with the established record of *Enterococcus* strains in food and probiotic applications [26,27,28], extending that evidence to a therapeutic infection model. The genomically confirmed absence of virulence determinants and antimicrobial resistance genes in *E. mundtii* CRL35 [64], combined with its food-grade origin from artisanal fermented foods [82,83], further supports its translational feasibility as a safe therapeutic candidate.

The multi-component nature of *E. mundtii* CRL35 CFS confers a theoretical advantage against resistance development. Unlike conventional antileishmanial drugs acting through specific molecular targets susceptible to mutation-driven resistance, CFS components exert lethal effects through multiple cellular targets, probably including direct membrane disruption, a mechanism inherently more difficult for parasites to circumvent [13,84]. The MCSI developed in this study, integrating five clinical parameters into a composite metric, offers a semi-quantitative tool that bridges qualitative visual assessments and quantitative parasitological measures, facilitating cross-study comparisons in chronic cutaneous infection models. In this context, the safety profile and administration routes of *E. mundtii* CRL35-based treatments make them promising candidates even for canine leishmaniasis, where reducing the animal reservoir through accessible and non-invasive interventions could contribute to human disease control through a One Health approach. Comparative studies with Leishmania species of epidemiological relevance, including *L. (V.) braziliensis* and *L. (V.) guyanensis*, would address whether CRL35-based treatments retain efficacy across species with distinct immunopathological profiles.

Together, these characteristics establish *E. mundtii* CRL35 as a validated experimental platform for bacteria-based therapeutics in parasitic diseases. Future studies can leverage the protocols developed here to identify the specific CFS bioactive components responsible for antileishmanial activity, enabling structure–activity relationship studies and rational optimization of metabolite production. The dual-route treatment design additionally provides a generalizable framework for evaluating other recognized as safe or antimicrobial-producing bacteria against cutaneous infections where local and systemic immunity both contribute to outcomes. The genomically confirmed absence of virulence determinants and antimicrobial resistance genes, combined with the food-grade origin of this strain, further supports its translational feasibility. Beyond these immediate applications, formulation engineering, including encapsulation strategies for oral delivery and hydrogel-based topical vehicles for sustained CFS release, could enhance therapeutic indices and patient adherence, establishing the foundation for future bioengineering approaches. Yet the interpretation of these results warrants consideration of several important constraints. The BALB/c/*L. (L.) amazonensis* model, while highly susceptible and well-characterized, may not fully recapitulate the immune dynamics of human ATL, where a more heterogeneous host response is observed. Additionally, other clinically relevant Leishmania species, particularly *L. (V.) braziliensis* and *L. (V.) guyanensis*, which predominate in certain endemic regions and exhibit distinct virulence profiles, were not evaluated. These species may display differential susceptibility to *E. mundtii* CRL35-based treatments due to variations in pathogenicity, tissue tropism, and host-parasite interactions. Furthermore, evaluation was limited to a single *L. (L.) amazonensis* strain (MHOM/BR/73/M2269); inter-strain variability among *L. (L.) amazonensis* isolates could influence treatment outcomes and generalizability. Similarly, validation in additional mouse strains, and alternative animal models would address this concern and strengthen the translational relevance of future preclinical investigations.

## 5. Conclusions

In summary, the present study demonstrates that *E. mundtii* CRL35, administered as a topically applied cell-free supernatant (CFS-T) or as viable bacteria given orally (LB-O), constitutes a safe and effective therapeutic strategy against cutaneous leishmaniasis caused by *L. (L.) amazonensis*, with parasitological and macroscopic outcomes superior to amphotericin B at the lesion site. Importantly, none of the *E. mundtii* CRL35-based treatments exacerbated infection-associated organ involvement or systemic biochemical alterations, in contrast to hepatotoxicity observed with amphotericin B. Together, these findings position *E. mundtii* CRL35-based strategies as safe, accessible, and effective alternatives for ATL treatment in resource-limited endemic settings, warranting further investigation through fractionation of bioactive CFS components, evaluation against additional Leishmania species, and comprehensive immunological profiling to elucidate the mechanisms underlying oral treatment efficacy.

## Figures and Tables

**Table 1:** Macroscopic Clinical Severity Index (MCSI): parameters and scoring criteria. ^a^ $A_{observed}$ is the measured lesion area for each animal and $A_{reference}$ is the reference lesion area corresponding to the average of the untreated control group in the experimental model. ^b^ Indicators of inflammation: perilesional erythema, perilesional edema, increased exudate volume, and friable or easily bleeding tissue. ^c^ Indicators of healing/re-epithelialization: reddish tissue coloration, raw flesh-like texture, moist appearance without pus, and dry appearance with crust formation.

Parameter	Score	Criteria
**Lesion size** **^a^**	0 to 5	The score was obtained using the following equation: $SizeScore=\frac{A_{observed}}{A_{reference}}\times 5$
**Wound edges**	0 to 4	0: well-defined 1: mild thickened 2: moderate thickened 3: elevated with notable thickening 4: damaged with necrosis
**Exudate type**	0 to 4	0: absent 1: serous 2: serosanguineous 3: sanguinolent 4: purulent
**Signs of inflammation** **^b^**	0 to 2	0: none 1: one or two signs 2: three or more signs
**Signs of healing** **^c^**	0 to −2	0: three or more signs 1: one or two signs 2: none

**Table 2:** Antileishmanial activity against *L. (L.) amazonensis* promastigotes, cytotoxicity toward RAW 264.7 macrophages, and selectivity indices of *Enterococcus* sp. cell-free supernatants. ID_50_, 50% inhibitory dilution (highest two-fold serial dilution producing ≥50% inhibition of parasite viability); CD_50_, 50% cytotoxic dilution (highest two-fold serial dilution producing ≥50% reduction in macrophage viability); SI, selectivity index (ID_50_/CD_50_). Antileishmanial activity is expressed as arbitrary units per milliliter (AU/mL) ± SEM. Higher AU/mL values indicate greater antileishmanial potency by reflecting the retention of inhibitory activity upon serial dilution. Higher SI values indicate greater selectivity toward parasites relative to host cells.

Cell-Free Supernatants	ID_50_ (AU/mL)	CD_50_ (AU/mL)	SI (ID_50_/CD_50_)
*E. mundtii* CRL35	1025 ± 167.7	10.81 ± 1.5	95
*E. faecium* IPE114	1048 ± 124.2	20.21 ± 5.9	52
*E. faecalis* IPE89	1162 ± 194.4	20.2 ± 9.9	58
*E. faecalis* IPE148	705.7 ± 109.9	8.54 ± 2.8	83

**Table 3:** Antileishmanial activity of *E. mundtii* CRL35 cell-free supernatant against intracellular *L. (L.) amazonensis* amastigotes. ID_50_, 50% inhibitory dilution; CD_50_, 50% cytotoxic dilution; SI, selectivity index (ID_50_/CD_50_). Values quantifying parasite viability following treatment were determined by indirect rescue assay. Data represent mean ± SEM of three independent experiments, each performed in triplicate.

Antileishmanial Activity of *E. mundtii* CRL35 CFS Against *L. (L.) amazonensis* Amastigotes		
ID_50_ (AU/mL)	CD_50_ (AU/mL)	SI (ID_50_/CD_50_)
270.2 ± 8.3	10.81 ± 1.5	25

## Data Availability

All data is available upon reasonable request to the authors.

## Abbreviations

The following abbreviations are used in this manuscript:

AmpB	Amphotericin B
ATL	American tegumentary leishmaniasis
AU/mL	Arbitrary units per milliliter
CD_50_	Half-maximal cytotoxic dilution
CFS	Cell-free supernatant
CFS-T	Cell-free supernatant, topical administration
ELISA	Enzyme-linked immunosorbent assay
ID_50_	Half-maximal inhibitory dilution
LAB	Lactic acid bacteria
LaAg	*Leishmania (L.) amazonensis* total antigen
LB-O	Live bacteria, oral administration
MCSI	Macroscopic Clinical Severity Index
qPCR	Quantitative polymerase chain reaction
SI	Selectivity index
WHO	World Health Organization
